# Fixed-Base Comb with Window-Non-Adjacent Form (NAF) Method for Scalar Multiplication

**DOI:** 10.3390/s130709483

**Published:** 2013-07-23

**Authors:** Hwajeong Seo, Hyunjin Kim, Taehwan Park, Yeoncheol Lee, Zhe Liu, Howon Kim

**Affiliations:** 1 Computer engineering, Pusan National University, Pusan 609-735, Korea; E-Mails: hwajeong@pusan.ac.kr (H.S.); moonmaker.k@gmail.com (H.K.); pth5804@pusan.ac.kr (T.P.); lycshotgunl@gmail.com (Y.L.); 2 Laboratory of Algorithmics, Cryptology and Security, University of Luxembourg, 6, Rue Richard Coudenhove-Kalergi, Luxembourg L–1359, Luxembourg; E-Mail: zhe.liu@uni.lu

**Keywords:** public key cryptography, elliptic curve cryptography, scalar multiplication, fixed-base comb method, window-NAF method, efficient implementation, embedded microprocessor

## Abstract

Elliptic curve cryptography (ECC) is one of the most promising public-key techniques in terms of short key size and various crypto protocols. For this reason, many studies on the implementation of ECC on resource-constrained devices within a practical execution time have been conducted. To this end, we must focus on scalar multiplication, which is the most expensive operation in ECC. A number of studies have proposed pre-computation and advanced scalar multiplication using a non-adjacent form (NAF) representation, and more sophisticated approaches have employed a width-*w* NAF representation and a modified pre-computation table. In this paper, we propose a new pre-computation method in which zero occurrences are much more frequent than in previous methods. This method can be applied to ordinary group scalar multiplication, but it requires large pre-computation table, so we combined the previous method with ours for practical purposes. This novel structure establishes a new feature that adjusts speed performance and table size finely, so we can customize the pre-computation table for our own purposes. Finally, we can establish a customized look-up table for embedded microprocessors.

## 1. Introduction

Elliptic curve cryptography (ECC) is a public-key cryptography based on the algebraic structure of elliptic curves over finite fields [1–3]. The use of elliptic curves in cryptography was suggested independently by Koblitz [4] and Miller [5] in 1985. The short key size and various crypto protocols are available in ECC, which enable secure and robust communications. However, scalar multiplication, which multiplies a secret scalar, *k*, with a point, *P*, on an elliptic curve, *E*( 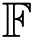
*_q_*), resulting in the point, *Q* ∈ *E*( 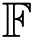
*_q_*), is too expensive to compute on embedded microprocessors. Various methods have been presented to boost the scalar multiplication ability. In particular, for a fixed point, *P*, we can take advantage of pre-computation tables for scalar multiplication, which were proposed in 1992 [6]. Using this method, the pre-computed points are immediately added to the results without explicit computation. In 1994, a further advanced method was proposed by Lim and Lee [7]. This presented a novel look-up table construction for flexible exponentiation. In 2005, Tsaur and Chou presented a non-adjacent form (NAF) representation based on a fixed-base comb method using direct doubling [8,9]. This allows the table to be efficiently computed in the case of multiple doubling. In 2012, Mohamed *et al.* presented a more sophisticated method using a width-*w* NAF representation and a different form of pre-computation table [10].

In this paper, we propose an efficient method for fixed-point scalar multiplication, enhancing the method of Mohamed *et al.* by constructing a novel look-up table structure. This structure generates consecutive zero-sequences more frequently, so we can compute the scalar multiplication with a smaller number of group addition operations. This characteristic is derived from observation of the *w*-NAF behavior and test secret scalars, *k*, given by the Blum-Blum-Shub random number generator, which is guaranteed by the National Institute of Standards and Technology (NIST) random number suite [11]. However, straight-forward implementation of our method does not show advantage, due to a large pre-computation table, so we mixed the previous method with ours in various combinations to meet the speed and size requirements. This novel structure has a new feature that can adjust size and speed more accurately, and this is unavailable in the previous method. With this hybrid method, we can construct various pre-computation tables for various purposes, such as the finely-tuned speed and size trade-off model. This method can be implemented on modern microprocessors, such as the ATmega, MSP and ARM series, which provide ROM more than at least 32 Kbytes. Furthermore, even an unknown point is efficiently computable by exploiting the proposed method. The detailed explanations of the unknown point are available in Appendix B. The following are the main contributions of this paper.

### Our Contributions

- We found zero occurrence characteristic of *NAF_w_* representation. This feature is useful to construct a pre-computation table with a high zero occurrence ratio.
- We presented a finely-tuned hybrid model, which can adjust size and speed performance more accurately. This can establish a customized look-up table for embedded microprocessors, by adjusting the program size or computation costs.
- We applied this method to an unknown point and show high performance enhancement by 12.7% compared to traditional *NAF_w_* representation.

The paper is organized as follows. In Section 2, we give an introduction to existing scalar multiplication methods, including those mentioned above. In Section 3, we introduce our proposed method, and in Section 4 we evaluate our proposal. Finally, we conclude our paper in Section 5.

## 2. Related Work

In this section, we explore scalar multiplication, which is one of the most expensive operations of elliptic curve cryptography. As shown in Algorithm 1, the inputs of the double-and-add algorithm are a random number, *k*, and a point, *P*, on an elliptic curve, *E*, defined over field, 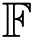
*_q_*, the output of this algorithm is another point, *Q*, on the same curve. Assuming the scalar, *k*, is 15, the binary representation of *k* is denoted by 0*b*1111; the hamming weights of *k* are four. Algorithm 1 requires three point doublings and four point additions, since the complexity of elliptic curve operations highly depends on the number of set or hamming weights, and the addition process can only be executed when the current scanned bit is non-zero. In order to further reduce hamming weights of the scalar, a number of encoding methods are proposed in succession, such as the work in our paper. Generally speaking, our method reduces the number of hamming weight by taking advantage of a pre-computing look-up table, especially for the fixed-base point case. In the following subsection, we introduce fixed-base methods in detail.


**Algorithm 1.** Double-and-add method using left-to-right binary method.
Input: *k* = (*k*_*t*-1_,…, *k*_1_, k_0_)2, *P* ∈ *E*(

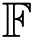
*_q_*).Output: *kP*.1.*Q* ← ∞.2.For *i* from *t* − 1 down to 0 do2.1 *Q* ← 2*Q*.2.2 If *k_i_* = 1, then *Q* ← *Q* + *P*.3.Return(*Q*)


### 2.1. (Fixed-Base) Scalar Multiplication Method

To describe scalar multiplication, we assume that #*E*( 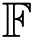
*_q_*) = *nh*, where *n* is prime and *h* is small (so *n* ≈ *q*), *P* and *Q* have order, *n*, and multipliers, such as *k*, are randomly selected integers from the interval, [1, *n* − 1]. The binary representation of *k* is denoted by (*k*_*t*–1_, …, *k*_2_, *k*_1_, *k*_0_)_2_, where *t* ≈ *m* = ⌈*log_2q_*⌉. If the point, *P*, is fixed and storage is available, point multiplication is accelerated by the exploiting pre-computation table. Our work is centered here to reduce hamming weight in the case of fixed-base.

#### 2.1.1. Non-Adjacent Form

If *P* is given by *P* = (*x, y;* ∈ *E*( 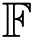
*_q_*), -*P* is represented as -*P* = (*x, -y*). Therefore, the subtraction of points on an elliptic curve can be executed using addition. This motivates the use of a signed digit representation, $k=\sum_{i=0}^{l=1}k_{i}2^{i}$, where *k_i_* ∈ {0, ±1}, and a particularly useful signed digit representation is the NAF [2].

#### 2.1.2. Window Method

The window method efficiently reduces the running time of scalar multiplication, using extra memory for pre-computation by window size. Thus, scalar multiplication time can be decreased by a window method that processes *w* digits of *k* at a time.

#### 2.1.3. Zero Occurrence Evaluation

Tables 1 and 2 show the relation of neighbor bit settings under *w*-NAF. We can find the *w*-NAF characteristic that, if a certain bit is set or reset, after the window size, the value of that bit has a high probability of being similar to the previous setting. For this reason, grouping the index by window size can obtain a setting with the same values. Finally, our structure efficiently separates zero and non-zero values by gathering the same values.

To improve its performance, the window method can be combined with NAF in a technique known as width-w NAF. *NAF_w_*(*k*) can be computed using Algorithm 2, where *k mods 2^w^* denotes the integer, *u*, satisfying *u* = *k*(*mod* 2*^w^*) and −2^*w*-1^≤ *u* < 2^*w*-1^. The digits of *NAF_w_*(*k*) are obtained by repeatedly dividing *k* by two, giving remainders, r, in [−2*^w−1^, 2^w−1^* − 1]. If *k* is odd and the remainder, *r* = *k mod* 2*^w^*, is chosen, then (*k* − r)/2 will be divisible by 2^*w*−1^, ensuring that the next *w* - 1 digits are zero. Using *NAF_w_*(*k*), the integer, *k*, is computed by window width, (*w*), from the left- to right-most bit, following Algorithm 3.


**Algorithm 2.** Computing the width-w NAF of a positive integer.
Input: Window width *w*, positive integer *k*.Output: *NAF_w_*(*k*).1.*i* ← 0.2.While *k ≥* 1 do2.1 If *k* is odd, then: *k_i_* ← *k* mods 2^*w*^, *k* ← *k* - *k_i_*2.2 Else: *k_i_* ← 0.2.3 *k*←*k*/2,*i*←*i* + 13.Return(*k_i_*_–1_, *k_i_*_–2_, …, *k*_1_, *k*_0_)



**Algorithm 3.** Window NAF method for point multiplication.
Input: Window width *w*, positive integer *k, P* ∈ *E*(

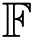
*_q_*).Output: *kP*.1.Use **Algorithm 2** to compute *w*-NAF 
$(k)=\sum_{i=0}^{l-1}k_{i}2^{i}$,2.Compute *P_i_* = *iP* for i ∈ {1, 3, 5,…, 2*^w^*^-1^ − 1}.3.*Q* ← ∞.4.For *i* from *l* − 1 down to 0 do4.1 *Q* ← 2*Q*.4.2 If *k_i_* ≠ 0, then4.2.1  If *k_i_*> 0 then *Q* ← *Q* + *P_ki_*.4.2.2  Else *Q* ← *Q − P_ki_*.5.Return(Q).


#### 2.1.4. Lim and Lee's Method

Let *R* be an n-bit exponent, for which we want to compute *g^R^* and divide the exponent, *R*, into *h* blocks, *R_i_*, for 0 ≤ *i* ≤ *h* − 1, of size $a=\left\lceil \frac{n}{h}\right\rceil$. We can subdivide each *R_i_* into *v* smaller blocks, *R_i,j_*, of size, $b=\left\lceil \frac{a}{v}\right\rceil$, for 0 ≤ *j* ≤ *v* − 1, as follows: *R_i_* = *R*_*i,v*-1_,…, *R*_*i*,1_, $R_{i,0}=\sum_{j=0}^{v-1}R_{i,j}2^{jb}$. Let *g*_0_= *g* and define *g_i_* as $g_{i}=g_{i-1}^{2^{a}}$ for 0 < *i* < *h*. Then, we can express *g^R^* as:

(1) $$g^{R}=\prod_{i=0}^{h-1}g_{i}^{R_{i}}=\prod_{j-0}^{v-1}\prod_{i=0}^{h-1}{\left(g_{i}^{2^{jb}}\right)}^{R_{i,j}}$$

Let *R_i_* = *e*_*i,a*-1_,…, *e*_*i*,1_, *e*_*i*,0_ be the binary representation of *R_i_*(0 ≤ *i* < *h*). Then, R_i,j_(0 ≤ *j* < *v*) is represented in binary as *R_i,j_*= *e_i_*,_j*b*+*b*−1_,…, *e*_*i,jb*+*k*_,… *e_i_*_,j_*_b+_*_1_,*e_i_*,*_jb_*. Therefore, Expression (2) can be rewritten as follows:

(2) $$g^{R}=\prod_{k=0}^{b-1}\;{\left(\prod_{j=0}^{v-1}\prod_{i=0}^{h-1}g_{i}^{2^{j^{b}}e_{i,jb+k}}\right)}^{2^{k}}$$

The following values are pre-computed and stored for all 1 ≤ *i* < 2*^h^* and 0 ≤ *j* < *v*. The index, *i*, is equal to the decimal value of *e*_*h*−1_,…, *e*_1_, *e*_0_.:

(3) $$G[0][i]=g_{h-1}^{e_{h-1}},g_{h-2}^{e_{h-2}},\ldots ,g_{1}^{e_{1}},g_{0}^{e_{0}},G[j][i]={\left(G[j-1][i]\right)}^{2^{b}}={\left(G[0][i]\right)}^{2^{j^{b}}}$$

Using the pre-computed values of Equation (3), the expression can be rewritten as Equation (4), where *I_j,k_* = *e*_*h*-1,*bj*+*k*_, …, *e*_1,*bj*+*k*_, *e*_0,*bj*+*k*_(0 ≤ *j* < *b*). This corresponds to the *k*-th bit column of the *j*-th block column. The computation of *g^R^* using Equation (4) is described in Algorithm 4.

(4) $$g^{R}=\prod_{k=0}^{b-1}\;{\left(\prod_{j=0}^{v-1}G[j][I_{j,k}]\right)}^{2^{k}}$$

**Algorithm 4.** Lim and Lee's method for exponentiation.
Input: Exponent *R*, *g*(Fixed element of *Z_n_*)Output: *g^R^*.1.*u* = Π_ki=_*_h_g^m_i_b^i^^*2.*v* = *u*3.For *w* = *h* − 1 to 1 by −13.1*u* = *u* × Π*k_i_* = *wg^m_i_b^i^^*3.2*v* = *v*×*u*4.Return(v).


#### 2.1.5. Tsaur and Chou's Method

Let *k* be an *l*-bit scalar represented in NAF, and we describe the table in two-dimensional form. Divide *k* into *h* × *v* blocks from top-to-bottom and, then, from right-to-left, where $h=\left\lceil \frac{l}{a}\right\rceil$. *k* can then be rewritten as Equation (5).

(5) $$k=c_{a-1},c_{a-2},\ldots ,c_{1},c_{0}=\sum_{l=0}^{a-1}c_{l}2^{lh}.$$

From right-to-left, the *h* × *a* blocks are then divided into *h* × *v* blocks, each of size, $b=\left\lceil \frac{a}{v}\right\rceil$.

Let *P*_0_= *P* and *P_j_* = 2^*hb*^*P*_*j*−1_= 2^*jhb*^*P* for 0 < *j* < *v*. Therefore, we can rewrite *kP* as Equation (6), where *c*_*jb*+*t*_= *e*_*h*-1,*jb*+*t*_,… *e*_1,*jb*+*t*_, *e*_0,*jb*+*t*_ is the NAF representation.

(6) $$\begin{array}{ll} & kP=c_{a-1},c_{a-2},\ldots ,c_{1},c_{0}P\\ =\sum_{l=0}^{a-1}c_{l}2^{lh}P & =\sum_{t=0}^{b-1}2^{th}\left(\sum_{j=0}^{v-1}c_{jb+t}2^{jhb}P\right). \end{array}$$

Suppose that the following values described in expression (7) are pre-computed and stored for all $1\leq s\leq \sum_{i=1}^{\left\lceil \frac{h}{2}\right\rceil }2^{h-2i+1}$ and 0 ≤ *j* ≤ *v* − 1, where *I_j,t_* is the decimal representation of *e*_*h*-1,*jb*+*t*_, …, *e*_1,*jb*+*t*_, *e*_0*jb*+*t*_.

(7) $$G[0][s]=e_{h-1}2^{h-1}P+e_{h-2}2^{h-2}P+\ldots +e_{0}P,G[j][s]=2^{hb}(G[j-1][s])=2^{jhb}G[0][s]$$

Using expression (7), *kP* can be rewritten as in Equation (8).

(8) $$kP=\sum_{t=0}^{b-1}2^{th}\left(\sum_{j=0}^{v-1}G[j][I_{j,t}]\right)$$

**Algorithm 5.** Tsaur and Chou's method for scalar multiplication.
Input: Positive integers *k*, *P* ∈ *E*(

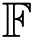
*_q_*).Output: *Q* = *kP*.1.*R*=∞2.For *k* = *b* - 1 to 0 by −1 do2.1 If *h* = 1, then2.1.1  *R* = 2*R*.2.2 Else2.2.1  Compute *R* = 2*^h^R* using **Algorithm 6**.2.3 For *j* = *v* - 1 to 0 by −1 do2.3.1  If (*e_h-1,bj+k_*, …, *e*_0_,*_bj+k_*)*_NAF_* > 0 then2.3.1.1   *R = R* + G[j] [*I*_j,k_]2.3.2 Else if (*e_h_*_-1_,*_bj+k_*, …, *e*_0_,*_bj+k_*)*_NAF_* < 0 then2.3.2.1  
$I_{j,k}^{'}=-{\left(e_{h-1,bj+k},\ldots ,e_{1,bj+k},e_{0,bj+k}\right)}_{NAF}$2.3.2.2  *R= R* − G[j] [
$I_{j,k}^{'}$]3.Return *R*



**Algorithm 6.** Sakai and Sakurai's method for direct doubling.
Input: A positive integer *r* such that *k* = 2*^r^* and *P* ∈ *E*(

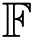
*_q_*).Output: *k* = 2*^r^P*.1.*A*_1_ = *x*_1_,. 
$B_{1}=3x_{1}^{2}+a$, *C*_1_ = -y_1_.2.For *i* = 2 to *r*.2.1 
$A_{i}=B_{i-1}^{2}-8A_{i-1}C_{i-1}^{2}.$2.2 
$B_{i}=3A_{i}^{2}+16^{i-1}a{\left(\prod_{j=1}^{i-1}C_{j}\right)}^{4}.$2.2 
$C_{i}=-8C_{i-1}^{4}-B_{i-1}(A_{i}-4A_{i-1}C_{i-2}^{2}).$3.Compute 
$D_{r}=12A_{r}C_{r}^{2}-B_{r}^{2}.$4.Compute 
$x_{2}r=\frac{B_{r}^{2}-8A_{r}C_{r}^{2}}{{\left(2^{r}\prod_{i=1}^{r}C_{i}\right)}^{2}}.$5.Compute 
$y_{2}r=\frac{8C_{r}^{4}-B_{r}D_{r}}{{\left(2^{r}\prod_{i=1}^{r}C_{i}\right)}^{3}}.$6.Return *x*_2_*_r_*, *y*_2_*_r_*



**Algorithm 7.** Mohamed, Hashim and Hutter's method for scalar multiplication.
Input: Positive integers *w,v,k* = (*k_l-1_*,…, *k*_1_, *k_0_*)*_NAFw_, P* ∈ *E*(

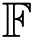
*_q_*).Output: *Q* = *kP*.1.
$a=\left\lceil \frac{l}{w}\right\rceil$, 
$b=\left\lceil \frac{a}{v}\right\rceil$2.Compute *G*[*0*] [*sd*], *G*[*j*] [*sd*] for all s ∈ {1,2, 2^2^, 2^3^,…, 2^*w*-1^}, 0 < *j* ≤ *v* − 1,*d* ∈ {1,3,5,…, 2*^w^*^-1^ − 1}.3.*Q* = ∞4.For *t = b* − 1 down to 0 do4.1 If *w* = 1, then4.1.1  *Q* = 2*Q*.4.2 Else4.2.1  Use **Algorithm 6** to compute *Q* = 2*^w^Q*.4.3 For *j* = *v* − 1 down to 0 do4.3.1  *I_j.t_*=(*k_jb+t,w_*_-1_,…, *k_jb+t_*,_0_)_NAFw_4.3.2  If *I_j,t_* > 0, then4.3.2.1   *Q* = *Q* + *G*[*j*] [*I_j,t_*].4.3.3  Else if *I_j,t_* < 04.3.3.1   *Q* = *Q − G*[*j*] [*−I_j,t_*].5.Return (*Q*).


#### 2.1.6. Direct Doubling Method

Sakai and Sakurai proposed a multi-doubling method for elliptic scalar multiplication. This reduced computational complexity by applying one constant inversion operation, regardless of the number of doubling. The complexity is given as (4r + 1)*M* + (4*r* + 1)*S* + *I*,, where *M, S* and *I* denote a multiplication, a squaring and an inversion in 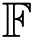
*_q_*, respectively.

#### 2.1.7. Mohamed, Hashim and Hutter's Method

This method represents the scalar, *k*, in width-*w* NAF. First, *k* is divided into $a=\left\lceil \frac{l}{w}\right\rceil$ blocks of equal size, *w. k* can then be written as follows:

(9) $$kP=K_{a-1}K_{a-2}\ldots K_{1}K_{0}=\sum_{d=0}^{a-1}K_{d}2^{dw}$$

where 0 ≤ *d* < *a*. Each block, *K_d_*, is then a column of *w* bits, and each block consists of *w* values. Hence, a block can be represented in *w* rows and is rewritten as *k_d,dw+i_*. For each element, *k_d,dw+i_*, the first subscript, *d*, indicates the column, whereas the second subscript, *dw* + *i*, indicates the exact bit index from width-*w* NAF (*k*). To simplify the notation in the following, *k_d,dw+i_* is written as *k_d,i_*. From right-to-left, *w* × *a* blocks are divided into *w* × *b* × *v* blocks, each of size, $b=\left\lceil \frac{a}{v}\right\rceil$, and rewritten as follows:

(10) $$kP=K_{a-1}K_{a-2}\ldots K_{1}K_{0}P=\sum_{j=0}^{v-1}\sum_{t=0}^{b-1}(K_{jb+t}2^{tw})2^{jbw}P=\sum_{t=0}^{b-1}2^{tw}\sum_{j=0}^{v-1}K_{jb+t}2^{jbw}P$$

where *K*_*jb*+*t*_= *k*_*jb*+*t,w*−1_… *k*_*jb*+*t*,0_ is in width-*w* NAF representation. The following values are pre-computed and stored for all *s* ∈ {1,2, 2^2^, 2^3^,…, 2^w−1^},0 < *j* ≤ *v* − 1 and *d* ∈ {1,3,…, 2^*w*−1^− 1}:

(11) $$\begin{array}{c} G[0][sd]=e_{w-1}2^{w-1}P+e_{w-2}2^{w-2}P+\ldots +e_{0}P=sdP\\ G[j][sd]=2^{wb}(G[j-1][sd])=2^{jwb}G[0][sd]=2^{jwb}sdP \end{array}$$

where the index, *sd*, is equal to the decimal value of (*e*_*w*−1_… *e*_1_*e*_0_). Therefore, *kP* can be rewritten as $kP=\sum_{t=0}^{b-1}2^{tw}\left(\sum_{j=0}^{v-1}G[j][I_{j,t}]\right)$, where *I_j,t_* is the decimal value of *k*_*jb*+*t*_,_*w*−1_… *k*_*jb*+*t*,0_.

## 3. (Proposed) Fixed-Base Comb with Window-NAF Method

This method represents the scalar, *k*, in the width-*w* NAF used for the previous approach. First, the scalar, *k*, having length (*l*), is divided into $a=\left\lceil \frac{l}{w}\right\rceil$ blocks of equal size, *w*, and *k* can be written as follows:

(12) $$kP=K_{a-1}K_{a-2}\ldots K_{1}K_{0}=\sum_{d=0}^{a-1}K_{d}2^{dw}$$

where 0 ≤ *d* < *a*. Then, each block, *K_d_*, is a column of *w* bits, and each block consists of *w*-bit elements; so a block can be represented in *w* rows and is rewritten as *k*_*d,dw*+*i*_. For each element, *k*_*d,dw*+*i*_, the first subscript, *d*, indicates the column, whereas the second subscript, *dw* + *i*, indicates the exact bit index from width-*w* NAF (*k*). The number of look-up tables is $z=\left\lceil \frac{a}{w}\right\rceil$. To simplify the notation in the following, *k*_*d,dw*+*i*_ is written as *k_d,i_* and rewritten as follows:

(13) $$kP=K_{a-1}K_{a-2}\ldots K_{1}K_{0}P=\sum_{t=0}^{z-1}\sum_{i=0}^{w-1}(K_{tw^{2}+i}2^{i})P$$

where *K*_*tw*^2^+*i*_= *k*_*tw*^2^+*w*(*w*−1),*i*_… *k*_*tw*^2^+*w,i*_*k*_*tw*^2^,*i*_ is in width −*w* NAF representation. The following values are pre-computed and stored for all *s* ∈ {1,2, 2^2^, 2^3^, …, 2^*w*-1^} and *d* ∈ {20, 2^*w*^,…, 2^*w*(*z*−1)^}.

(14) $$\begin{array}{r} G[0][sd]=sdP=\{e_{0}2P+e_{w}2^{w}P\ldots +e_{w(w-1)}2^{w(w-1)}P\}+\\ 2^{w^{2}}\{e_{w^{2}}2P+e_{w^{2}\cdot w}2^{w}P\ldots +e_{w^{2}\cdot (w(w-1))}2^{w(w-1)}P\}+\\ \ldots +2^{w^{2}(z-1)}\{e_{w^{2}(z-1)}2P+e_{w^{2}(z-1)\cdot w}2^{w}P\ldots +e_{w^{2}(z-1)\cdot (w(w-1))}2^{w(w-1)}P\}\\ G[i][sd]=2(G[i-1][sd])=2^{i}G[0][sd]=2^{i}sdP \end{array}$$

where the index, *sd*, is equal to the decimal value of (*e*_*w*^2^(_*_z−_*_1)_…*e*_*w*^2^_·_*w*_*e*_*w*^2^_ …*e*_*w*_*e*_0_). Therefore, *kP* can be rewritten as $kP=\sum_{i=0}^{w-1}2^{i}\left(\sum_{t=0}^{z-1}G[i][I_{i,t}]\right)$, where I_i_*_,t_* is the decimal value of *k*_*zw*^2^+*w*(*w*-1)_*_,i_*…. *k*_*zw*^2^,*i*_) *_NAFw_*.


**Algorithm 8.** (Proposed) Fixed-base comb with Window-NAF method for scalar multiplication.
Input: Positive integers *w,v,k* = (*k_l_1_*, …,*k*_1_, *k*_0_)_NAF_*_W_*, *P* ∈ *E*(

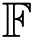
*_q_*).Output: *Q* = *kP*.1.
$a=\left\lceil \frac{l}{w}\right\rceil$, 
$z=\left\lceil \frac{a}{w}\right\rceil$2.Compute *G*[0][*sd*], *G*[*i*][*sd*] for all s ∈ {1, 2, 2^2^, 2^3^,…, 2^*w*-1^}, 0 < *i ≤* w-1, d ∈ {2^0^, 2^w^,…, 2^w(a-1)^}.3.*Q* = ∞4.For *i* = 0 to *w* − 1 do4.1 *Q* = 2*Q*.4.2 For *t = z − 1* down to 0 do4.2.1  *I_i,t_*=(*k_zw^2^+w_*_(_*_w_*_-1)_,*_i_*… *k_zw_*2)_NAFW_.4.2.2  If *k_zw_*_2+_*_w_*_(_*_w_*_-1)_ > 0, then4.2.2.1   *Q* = *Q* + *G*[*i*] [*I_i,t_*].4.2.3  Else if *k_zw_*_2+_*_w_*_(_*_w_*_-1)_ < 04.2.3.1   *Q* = *Q* − *G*[*i*] [−*I*_*i,t*_].5.Return (*Q*).

### 3.1. Comparison of Pre-Computation Table Structures

**Figure 1.**
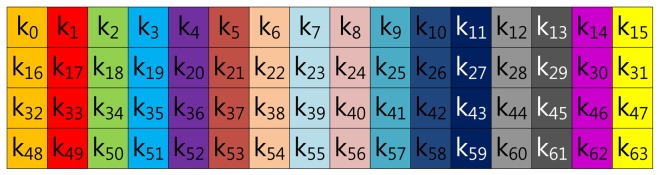
Look-up table structure of Lim and Lee's method in a block form.

In this section, we demonstrate fixed-base scalar multiplication in a block form to allow a comparison of the table structures. In the example, we use a 64-bit scalar value, *k*, and a look-up table with width-4 index. To illustrate the look-up table index more vividly, we use the same color for the same group elements. Figure 1 shows the structure of Lim and Lee's method when the width of the block index, (a), is set to 16. In the Figure, elements are grouped in this order: (*k*_0_, *k*_16_, *k*_32_, *k*_48_), …, (*k*_15_, *k*_31_, *k*_47_, *k*_63_).

**Figure 2.**
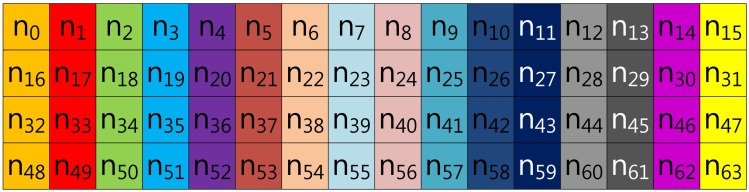
Look-up table structure of Tsaur and Chou's method in a block form for 2NAF.

In the case of Tsaur and Chou's method described in Figure 2, the look-up table has the same structure as in Lim and Lee's method, so elements are grouped in this order: (*n*_0_, *n*_16_, *n*_32_, *n*_48_), …, (*n*_15_, *n*_31_, *n*_47_, *n*_63_). However, the scalar value (*k*) is represented in *NAF*_2_(*n*) to generate frequent consecutive zero sequences, which can reduce the overhead of group addition.

**Figure 3.**
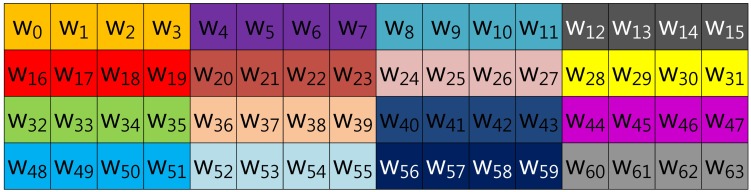
Look-up table structure of Mohamed, Hashim and Hutter's method in a block form for 4NAF.

In the case of Mohamed, Hashim and Hutter's method described in Figure 3, the scalar value (*k*) is represented in window-NAF (*w*), which replaces the adjacent set values to zero values by window size. The look-up table index is grouped in incremental order by window size, and elements represented in *w*-NAF are grouped in this order: (*w*_0_, *w*_1_, *w*_2_, *w*_3_), …, (*w*_60_, *w*_61_, *w*_62_, *w*_63_). This structure is efficiently reducing table size, because within window size, only one element can have a value.

**Figure 4.**
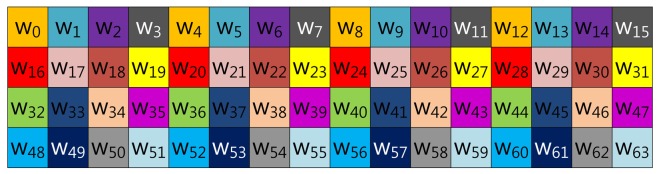
Look-up table structure of the proposed method in a block form for 4NAF.

Our proposed method described in Figure 4 represents the scalar value (*k*) in window-NAF (*w*). Mohamed *et al.* grouped values in incremental order of index. On the other hand, we use a different look-up table structure, following the characteristic that the set value in one position will affect the following value's bit setting. For window size, *w*, the *a*-th and the (*a* + *w*)th sub-windows, e.g., in the case of *w* = 4, (*w*_0_, *w*_4_), (*w*_2_, *w*_6_), (*w*_3_, *w*_7_) and (*w*_4_, *w*_8_), exhibit a strong interrelationship. If one value is set, the other has a high probability of being set, and the opposite case shows same results. In Table 1, we give a test on this features by testing all cases. This characteristic can be used to construct a look-up table with a more frequent number of zero occurrences, so our method constructs a table in this order: (*w*_0_, *w*_4_, *w*_8_, *w*_12_), …, (*w*_51_, *w*_55_, *w*_59_, *w*_63_).

### 3.2. Size Optimized Method

**Figure 5.**
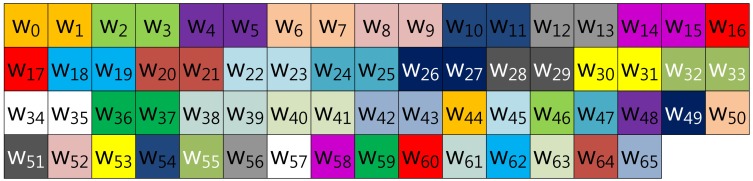
Look-up table structure of (size-optimized) the proposed method in a block form for 3NAF (version 1).

**Figure 6.**
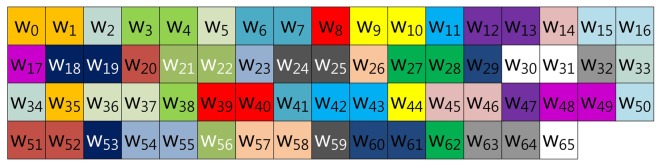
Look-up table structure of the (size-optimized) proposed method in block form for 3NAF (version 2).

**Figure 7.**
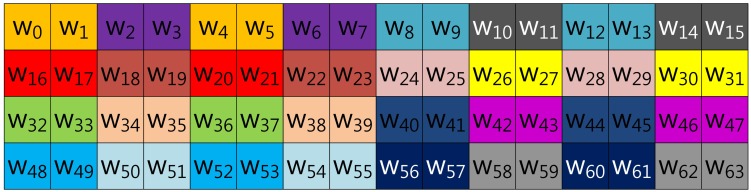
Look-up table structure of the (size-optimized) proposed method in block form for 4NAF (version 3).

**Figure 8.**
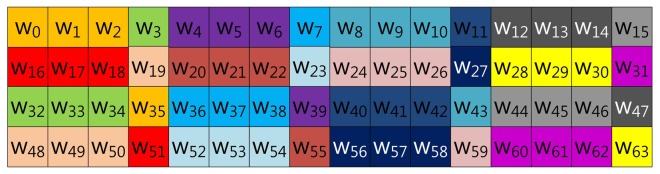
Look-up table structure of the (size-optimized) proposed method in block form for 4NAF (version 4).

The size-optimized model has a pre-computation table combining the proposed method and that of Mohamed *et al.* Therefore, the method constructs the table structure with consecutive elements and elements in distance. The consecutive elements represented in NAF form do not have consecutive values, so the number of cases in the table is smaller than the table constructed with elements in distance. Unlike the consecutive elements, an element selected in distance shows higher zero occurrence, but the table size is much larger than consecutive elements. For this reason, the method combining both table structures reduces the table size, while it degrades speed performance. However, reduction of speed performance is minor, compared to the huge reduction of table size. Examples of 3NAF and 4NAF are illustrated in Figures 5, 6, 7 and 8, respectively. In Figure 5, two elements are selected from a consecutive index, and one element is chosen in distance. The elements are grouped in this order: (*w*_0_, *w*_1_, *w*_44_), (*w*_22_, *w*_23_, *w*_45_) …, (*w*_20_, *w*_21_, *w*_64_), (*w*_42_, *w*_43_, *w*_65_). To compute this structure, elements are added to a result, and then, the result is doubled twice.

In Figure 6, a different 3NAF look-up table, version 2, is illustrated. Elements are grouped in the same structure of that of the previous one, but the index is mixed. This structure combines the elements by window size, so it can group similar characteristic elements, but this structure requires a doubling process three times, because the elements are grouped by window size: three. In this model, the elements are grouped in this order: (*w*_0_, *w*_1_, *w*_35_), (*w*_2_, *w*_33_, *w*_34_) …, (*w*_32_, *w*_63_, *w*_64_), (*w*_30_, *w*_31_, *w*_65_).

In Figure 7, the 4NAF look-up table, version 3, is illustrated. The elements are grouped in two structure, which are placed in a window size distance, and the inner structures of this follow Mohamed *et al.* To compute scalar multiplication, a grouped element is added, and then, doubling is conducted twice in a row; then, addition is computed. In the example, the elements are grouped in this order: (*w*_0_, *w*_1_, *w*_4_, *w*_5_), (*w*_2_, *w*_3_, *w*_6_, *w*_7_) …, (*w*_56_, *w*_57_, *w*_60_, *w*_61_), (*w*_58_, *w*_59_, *w*_62_, *w*_63_).

In Figure 8, a different 4NAF look-up table, named version 4, is introduced. The elements are grouped in two structures, and one inner structure follows Mohamed *et al.* The other index is placed in distance. This shows a size optimized model, because three elements are consecutively grouped, so this has a small number of table cases. However, compared to version 3, this model needs to compute a doubling process four times. In the example, the elements are grouped in this order: (*w*_0_, *w*_1_, *w*_3_, *w*_35_), (*w*_3_, *w*_32_, *w*_33_, *w*_34_) …, (*w*_31_, *w*_60_, *w*_61_, *w*_62_), (*w*_28_, *w*_29_, *w*_30_, *w*_63_).

### 3.3. Hybrid Method for Fine Tuning

The hybrid method combines two methods, including our size-optimized or speed-optimized model and that of Mohamed *et al.* The advantage of a combined model is in generating a fine-tuned look-up table, which can adjust speed and size accurately, because both of them have a trade-off relation. By adjusting the look-up table size property, we can generate a proper look-up table more efficiently and accurately. This is based on the previous observation that Mohamed *et al.* provided a small pre-computation table with low zero occurrence and ours provides a big pre-computation table with high zero occurrence. Therefore, if we represent the table as Mohamed *et al.*, that part shows low zero occurrence and a small table, and if we use the proposed table, this part shows high zero occurrence and a big table. Finally, we draw a trade-off relation by representing a table with the finely-tuned method. This relation is available in Tables 3 and 4.

**Figure 10.**
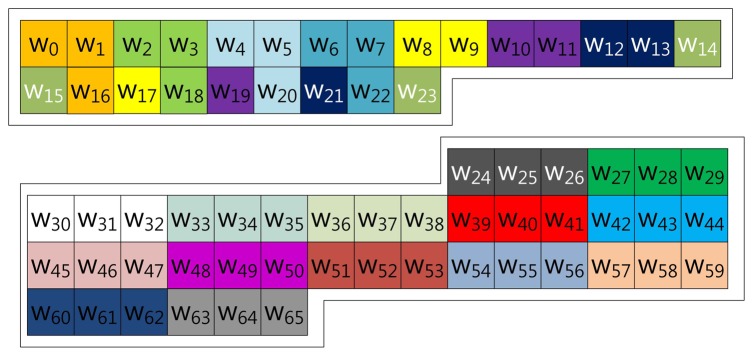
Look-up table structure of the (hybrid) proposed method in block form for 3NAF (version 1).

In Figure 10, a combined look-up table structure is described. From *w*_0_ to *w*_23_, 3NAF version 1 is used, and the remaining part follows Mohamed *et al.* The number of elements for each structure model can be adjusted. To compute this model, the addition of Mohamed *et al.* elements is computed, and then doubling is conducted once. Afterward, then, our size-optimized elements are added, and then direct doubling in a width of two is conducted.

**Figure 11.**
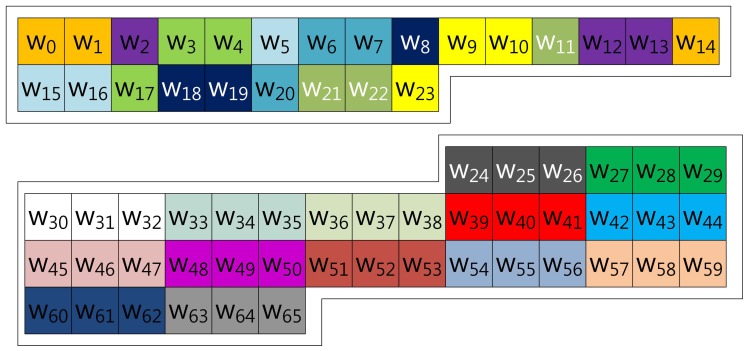
Look-up table structure of the (hybrid) proposed method in block form for 3NAF (version 2).

In Figure 11, the hybrid model of 3NAF version 2 is introduced. To compute this model, addition of Mohamed *et al.*'s and our model's elements are conducted, and then, direct doubling in a width of three is conducted for both structures. For this reason, more simple computation is available than the version 1 model.

**Figure 12.**
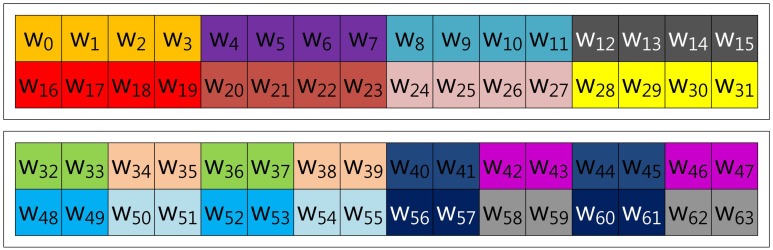
Look-up table structure of the (hybrid) proposed method in block form for 4NAF (version 3).

In Figure 12, the hybrid model of 4NAF version 3 is introduced. The version 3 model is structured in a width of two elements. To compute scalar multiplication, direct doubling in a width of two is conducted once, and then, our elements are added after. Then, direct doubling in a width of two is conducted.

**Figure 13.**
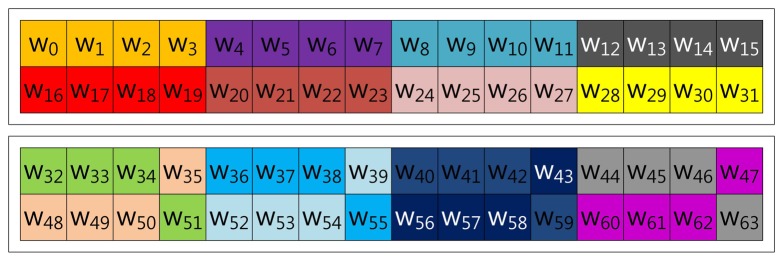
Look-up table structure of the (hybrid) proposed method in block form for 4NAF (version 4).

In Figure 13, the hybrid model of 4NAF version 4 is introduced. This model is conducted with direct doubling in a width of four. Compared to version 3, this model consumes a small-sized look-up table.

## 4. Results

In this section, we evaluate the proposed method in terms of its look-up table size and computation speed for practical performance evaluation.

### 4.1. Random Number Generator for Pair Evaluation

Random numbers are needed to evaluate the methods of generating the secret value (*k*) that is used to construct the look-up tables. For pair comparison, we choose a high-entropy random number algorithm (Blum-Blum-Shub) that is suited to the NIST random number suite. The difficulty of this pseudo-random number generator (PRNG) is based on integer factorization. When the prime is chosen appropriately, predicting the random patterns is at least as difficult as factoring prime. For this reason, using the output from this PRNG is suitable for pair comparison of computation.

### 4.2. Zero Occurrence of Look-up Table

Table 5 shows the percentage of zero occurrences under various conditions, such as the representation and window size. Lim and Lee use a normal representation, and the other methods are represented in NAF form. In each case, our proposed method shows better performance than previous published works. One interesting result is that the method of Mohamed *et al.* has a lower performance than that of Tsaur and Chou, but this becomes obvious after referring to the experimental results in this section. Within a given window size, values repeat periodically with high probability. Therefore, the model of Mohamed *et al.* has a low probability of having a zero sequence index for a given window size. On the other hand, Tsaur and Chou's method has no relation to the index of the look-up table, but shows higher performance.

Our model is perfectly suited to the characteristics of *w*-NAF, exhibiting a high zero occurrence. We also provide a size-optimized model, because our model requires a huge amount of storage for the look-up table. This model is properly adjusted for demand on speed and size.

**Figure 14.**
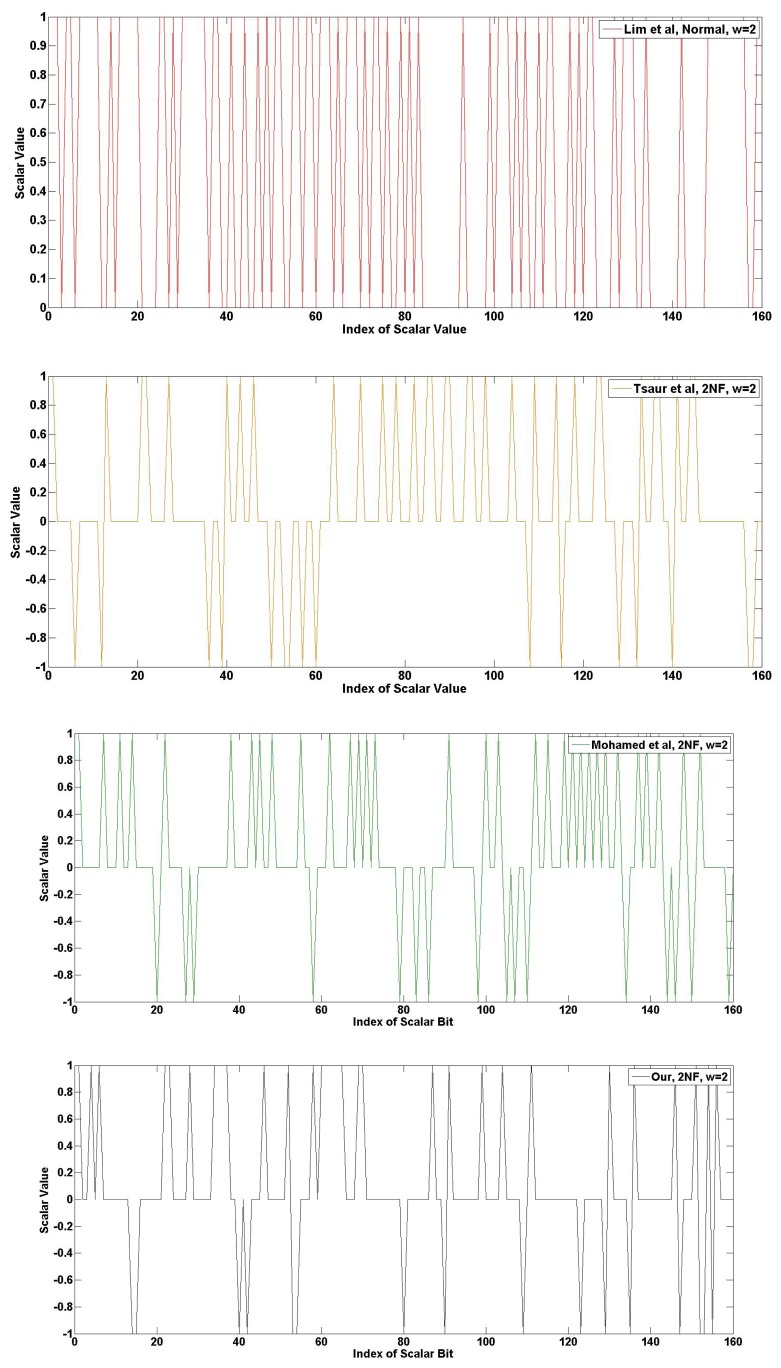
Frequency of scalar value represented in 2NAF.

**Figure 15.**
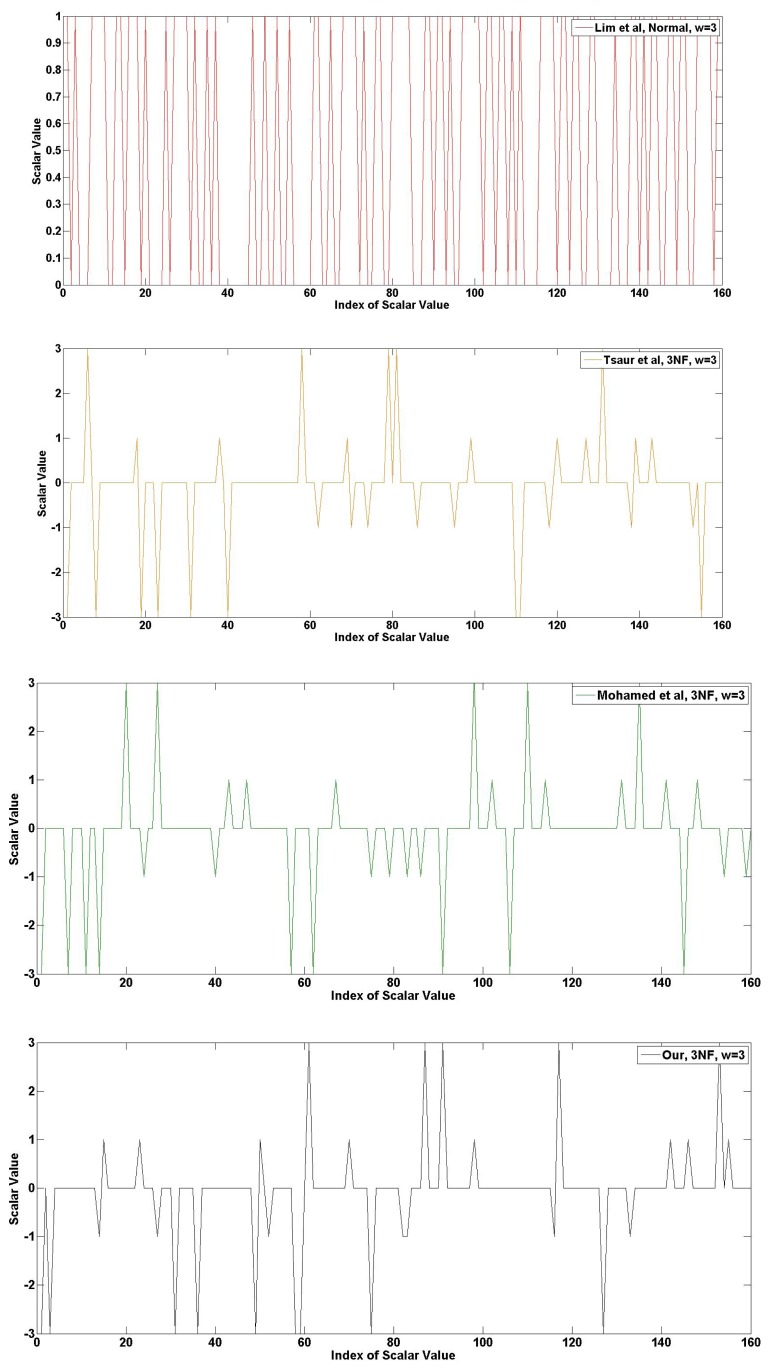

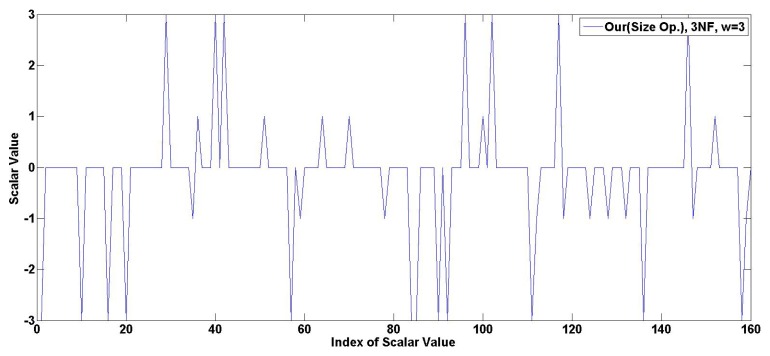
Frequency of scalar value represented in 3NAF.

**Figure 16.**
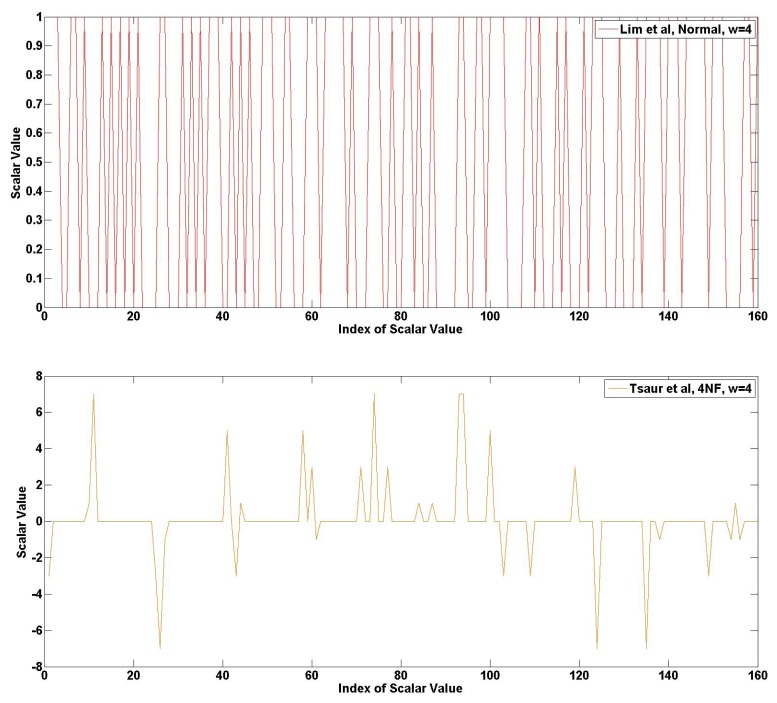

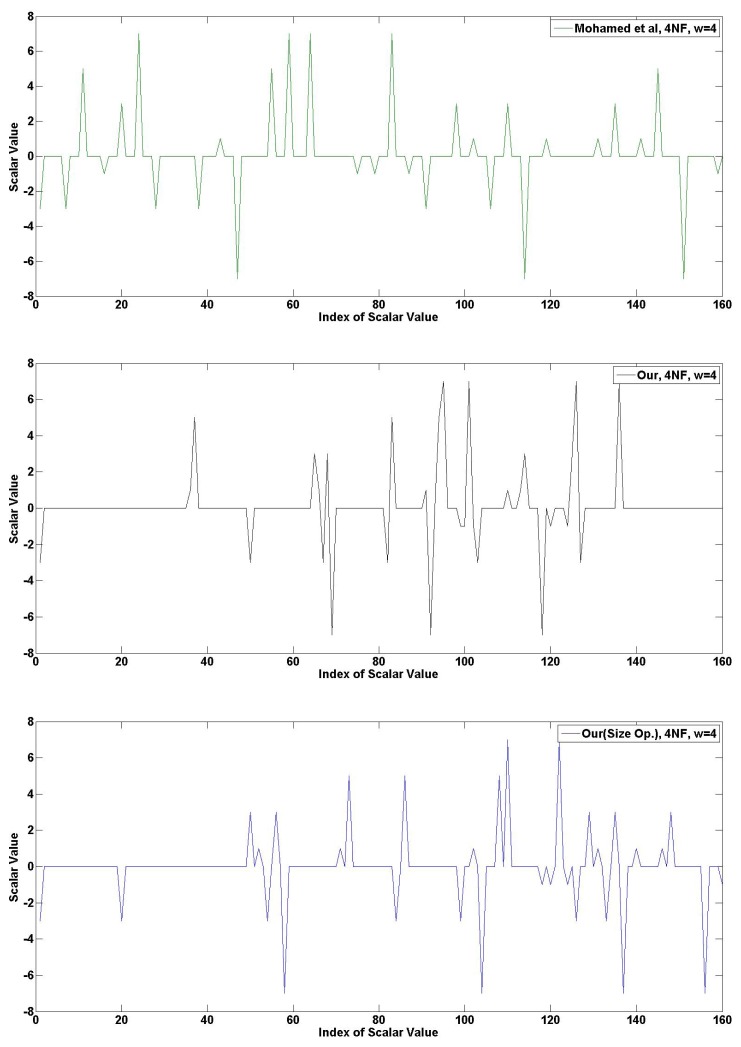
Frequency of scalar value represented in 4NAF.

The following Figures 14, 15 and 16 show the frequency of the scalar value in the case of 160-bits. The graphical results are generated from the random scalar value derived from Blum-Blum-Shub. In Figure 14, the frequency of 2NAF representation is described. The red graph is Lim *et al.*, and the value range is between one and zero, because this method does not use NAF form. The line in the graph is densely placed. The yellow graph is Tsaur *et al.*, and the value range is between one and minus one after transformation into 2NAF form. Compared to Lim *et al.*, many points are placed in zero value, and this generates consecutive zero values. The green and black graphs are Mohamed *et al.* and ours. The consecutive zero occurrences in our graph happen frequently, so this represents the efficiency of our method.

In Figure 15, the frequency of 3NAF representation is described. The red graph, Lim *et al.*, has high frequency, so it is hard to find zero occurrence. Unlike Lim *et al.*, other methods show a sparsely drawn graph. After 3NAF transformation, values are placed between three to minus three. At a glance, all representations appear to have a similar frequency. However, Table 5 shows that our method presents higher zero occurrences than any other methods.

In Figure 16, the frequency of 4NAF representation is described. This example describes the strength of our method vividly. The red graph, Lim *et al.*, obtains a higher frequency, so this has a high hamming weight. In the case of Tsaur *et al.*, there are low frequencies and many consecutive zeros. In the case of Mohamed *et al.*, the frequency is much higher than Tsaur *et al.*, so its hamming weight is higher than Tsaur *et al.* Finally, our method shows an impressive consecutive zero array. Furthermore, a high frequency is gathered in some regions. For this reason, this method is more efficient than other methods.

### 4.3. Size of Look-up Tables

In this section, we consider the size of the look-up tables, and detailed table size is described in Table 6. Lim and Lee's method has the smallest look-up table, and this can compute a storage size of (2^*w*^ − 1) × *t*, where *t* is the size of a point (in the case of 160-bit, 40 bytes are used, 20 for the x-axis and 20 for the y-axis), because elements consist of two values, zero and one. In the case of Tsaur and Chou's method, the number of elements for w-NAF is 2(*^w-^*^1)^ + 1, so the table size is ((2^(*w*-1^) + 1)^(*w*-1)^(2^(*w*-2)^ + 1)) × *t*. Mohamed *et* al.'s method has a smaller look-up table than that of Tsaur and Chou, because elements do not exist consecutively within each window size, due to the characteristics of *w*-NAF; so, the table size is *w* × 2^(*w*-2)^ × *t*. Our speed-optimized method is equivalent to Tsaur and Chou's method, because elements in the look-up table are randomly chosen, meaning that we should compute all cases. To reduce the size, while keeping a high zero occurrence, we provide a size-optimized model that has a high probability of zeros with reduced storage size. The size-optimized model combines our speed-optimized model and that of Mohamed *et al.*, so the table size estimation follows both methods partly, and the total table size is ((*w* − 1) × 2^(*w*-2)^ × (2^(*w*-1)^ + 1) + 2^(*w*-2)^) × *t*.

### 4.4. Computational Efficiency

Table 7 shows the performance results in terms of size and speed factors. For size, we calculate table size depending on the number of points. For speed, we estimated complexity from the number of group operations. In the table, we evaluate two cases, one using a partial look-up table and the other using the full look-up table for fixed point computation. The benefit of full computation is that all doubling computations are removed. Compared to Mohamed *et al.*, our speed-optimized method exhibits the best performance, due to the large number of zero occurrences, but it suffers from having a large look-up table. On the other hand, Mohamed *et al.*'s method has a small look-up table, but suffers from poor performance, due to the small number of zero occurrences. In the size-optimized model, it provides a proper zero occurrence probability with a much smaller table size than the speed model.

However, in Table 7, we compared performance unfairly, because we did not evaluate performance in the same look-up table size. Computation with a larger look-up table shows higher speed performance than a method with a small look-up table, because in the case of fixed-base point multiplication, a bigger look-up table ensure less point additions, and this directly affects faster scalar multiplication. For clarity, we further evaluate our performance in the same look-up table size and calculate overhead, generalized in multiplication, which is derived from Table 8, describing the relation of each operation in an affine coordinate. There are a number of operation dependencies, so we set our coordinate as affine and multiplication and squaring, and inversion is implemented in the basic method. Additionally, we refer to these basic relations from [2]. First, overheads of finite field squaring and inversion are re-written in the overhead of multiplication. Afterwards, then, group operations, overheads of addition, doubling and direct doubling are re-written in the overheads of multiplication.

In Table 9, we evaluate our model and that of Mohamed *et al.* under the same condition. Our size-optimized model (version 2) has a 12.3 Kbyte look-up table. Compared to the 4NAF model (partial table) our method outperforms in terms of size and speed factors. However, the 3NAF model (full table) shows much higher performance than ours with small size overheads. For this reason, we cannot confidently say that our method is faster than that of Mohamed *et al.* under the same condition. However, through this evaluation, we found that our method can be used for size and speed tuning techniques. We named it the hybrid method.

### 4.5. Hybrid Model Performance Evaluation

**Figure 17.**
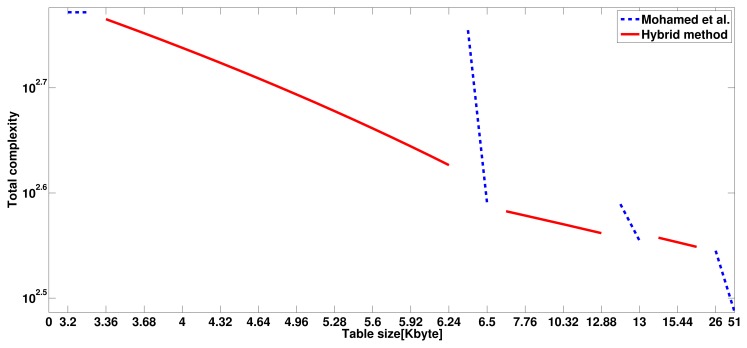
Performance of hybrid model using fine-tuned features.

Our hybrid model is developed during evaluation of the previous models to overcome the drawbacks of the proposed method. Tables 3, 4 and 10 describe the performance in size and speed. The most interesting feature is that we can adjust performance more finely. In Table 9, using Mohamed *et al.*'s method, there is no table model in 3.2∼6.4 and 6.5∼12.8 Kbyte. However, the hybrid model can tune the performance in terms of size and speed and provide various size models, including 3.2, 3.36, … 11.6 and 12.9 Kbytes. Various table models generate finely-tuned table structure for user's purposes. However, the hybrid model using 4NAF is not useful, because zero occurrence of 4NAF is far lower than 3NAF, so even 4NAF has a large table, but shows low speed performance. In Figure 17, a finely-tuned look-up table is depicted. In the figure, we found that with the blue dotted line, the method of Mohamed *et al.* is coarsely-tuned, so there is a large gap between each look-up table. However, our hybrid model can finely tune and fill gaps between each table, which is coarsely-tuned. Finally, we can complete the smooth graph, which means we can adjust our performance more accurately and finely.

## 5. Conclusions

Recently released novel scalar multiplication by Mohamed *et al.* reduced the number of addition operations using *w*-NAF and a novel look-up table structure. However, this method cannot carefully adjust their table structures in terms of speed and size matters, so this does not provide flexibility for selecting table structure, depending on different purposes. In this paper, we presented a novel method for fixed-point scalar multiplication. The new structure is firstly derived from *w*-NAF characteristics observed in this paper. However, our method shows similar performance when it is tested under the same look-up table size. However, constructing our table in various ways, we found one interesting features that can be used for speed and size performance adjustment. Finally, we presented a novel fine-tuned look-up table structure, which can provide more accurate and fine adjustment than previous methods. This idea can be applied to other combinations using many other table structures for the finely-tuned table model. This method can provide more flexible look-up tables for embedded microprocessors. Furthermore, the proposed method is straight-forwardly implementable on an unknown point, and this shows high performance enhancement compared to traditional *NAF_w_* methods.

## Figures and Tables

**Figure 9.**
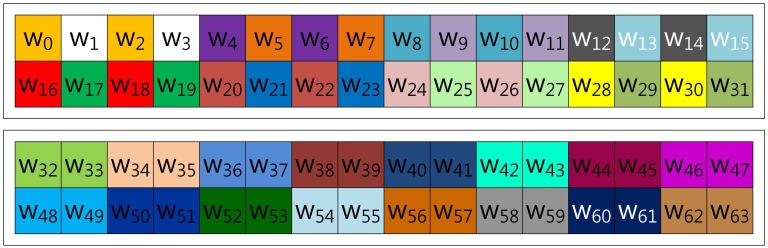
Look-up table structure of the (hybrid) proposed method in block form for 2NAF (speed-optimized model).

**Figure 18.**
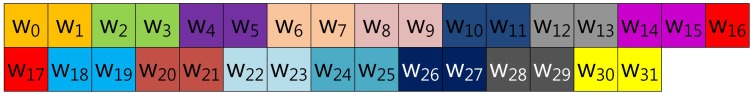
Look-up table structure of the traditional 2NAF method in block form.

**Figure 19.**
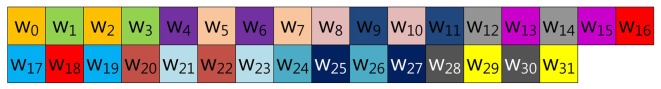
Look-up table structure of the proposed 2NAF method in block form.

**Table 1.** Relation of neighbor bit set in *w*-non-adjacent form (NAF) representation; we tested all cases for evaluation, so occurrence means number of cases.

**Form**	**Set**	**Being set**	**Occurrence**	**Prob (%)**
2NAF	1st	3rd	16	**67**
		4th	8	33

	2nd	3rd	0	0
		4th	8	**100**

3NAF	1st	4th	128	**36**
		5th	128	**36**
		6th	96	28

	2nd	4th	0	0
		5th	64	**50**
		6th	64	**50**

	3rd	4th	0	0
		5th	0	0
		6th	32	**100**

4NAF	1st	5th	262,144	**53**
		6th	131,072	27
		7th	65,536	13
		8th	32,768	7

	2nd	5th	0	0
		6th	131,072	**57**
		7th	65,536	29
		8th	32,768	14

	3rd	5th	0	0
		6th	0	0
		7th	65,536	**67**
		8th	32,768	33

	4th	5th	0	0
		6th	0	0
		7th	0	0
		8th	32,768	**100**

**Table 2.** Relation of neighbor bit reset in *w*-NAF representation; we tested all cases for evaluation, so occurrence means number of cases.

**Form**	**Reset**	**Being reset**	**Occurrence**	**Prob (%)**
2NAF	1st	3rd	24	**55**
		4th	20	45

	2nd	3rd	24	40
		4th	36	**60**

3NAF	1st	4th	448	**35**
		5th	416	33
		6th	400	32

	2nd	4th	576	32
		5th	608	**34**
		6th	624	**34**

	3rd	4th	704	**34**
		5th	672	32
		6th	720	**34**

4NAF	1st	5th	491,520	**30**
		6th	376,832	22
		7th	385,024	23
		8th	421,888	25

	2nd	5th	491,520	20
		6th	638,976	26
		7th	647,168	26
		8th	684,032	**28**

	3rd	5th	622,592	22
		6th	638,976	22
		7th	778,240	27
		8th	815,104	**29**

	4th	5th	688,128	23
		6th	704,512	23
		7th	778,240	25
		8th	880,640	**29**

**Table 3.** Performance evaluation for hybrid model in the case of our speed-optimized 2NAF; the bit section indicates the ratio of bits using our method and that of [10], respectively. Non-zero P: non-zero point, SP: speed optimized.

**Bit**		**Non-zero P**	**Total**	**Size**
**[10]**	**Here**	**Sp**	**Sp**	[**KB**]
160	0	52.88	591.46	3.2
156	4	52.05	582.60	3.36
152	8	51.23	573.74	3.52
148	12	50.40	564.88	3.68
144	16	49.58	556.02	3.84
140	20	48.76	547.17	4
136	24	47.93	538.31	4.16
132	28	47.11	529.45	4.32
128	32	46.28	520.59	4.48
124	36	45.46	511.73	4.64
120	40	44.64	502.88	4.8
116	44	43.81	494.02	4.96
112	48	42.99	485.16	5.12
108	52	42.16	476.30	5.28
104	56	41.34	467.44	5.44
100	60	40.52	458.59	5.6
96	64	39.69	449.73	5.76
92	68	38.87	440.87	5.92
88	72	38.04	432.01	6.08
84	76	37.22	423.15	6.24
80	80	36.4	414.3	6.4

**Table 4.** Performance evaluation for hybrid model in the case of 3NAF; versions 1 (V1) and 2 (V2) have the same table size. The bit section indicates the ratio of bits using our method and that of [10], respectively. Non-zero P: non-zero point.

**Bit**		**Non-zero P**		**Total**		**Size**
**[10]**	**Here**	**V1**	**V2**	**V1**	**V2**	[**KB**]
162	0	33.38	33.38	382.5	389.5	6.5
150	12	33.12	32.77	378.9	386.73	7.8
138	24	32.86	32.16	375.32	383.96	9
126	36	32.6	31.55	371.74	381.19	10.3
114	48	32.35	30.93	368.15	378.42	11.6
102	60	32.09	30.32	364.56	375.65	12.9
90	72	31.83	29.71	360.98	372.88	14.2
78	84	31.57	29.1	357.39	370.11	15.4
66	96	31.31	28.49	353.80	367.34	16.7

**Table 5.** Average percentage (%) of zero occurrences for a 160-bit scalar, tested using random number vectors from Blum-Blum-Shub (**Lim and Lee use a normal representation**), SP: speed-optimized model; V(1,3): size-optimized model in the case of version 1 and 3; V(2,4): size optimized model in the case of version 2 and 4.

**Form**	**[7]**	**[8]**	**[10]**	**SP**	**V(1,3)**	**V(2,4)**
2NAF	24.20	44.70	33.90	**50.20**	-	-
3NAF	11.59	48.98	38.20	**50.16**	46.54	44.64
4NAF	6.35	40.38	18.43	**53.33**	44.10	44.57

**Table 6.** Size (Kbyte) of the look-up table in the case of a 160-bit scalar (**Lim and Lee's method uses a normal representation**). Sp: speed optimized version; V(1,3): size optimized version 1 and 3; V(2,4): size optimized version 2 and 4.

**Form**	**[7]**	**[8]**	**[10]**	**Sp**	**V(1,3)**	**V(2,4)**
2NAF	0.12	0.16	0.08	0.16	-	-
3NAF	0.28	3.00	0.24	3.00	**0.88**	**0.88**
4NAF	0.60	145.80	0.64	145.80	**4.48**	**5.44**

**Table 7.** Evaluation result of fixed-base scalar multiplication, where *A*, *D* and *DD*(*w*) denote addition, doubling and direct doubling (width), respectively. Computation cost excludes a cost for look-up table construction, which is pre-computable, so the cost mainly includes group addition and doubling.

**Scheme**	**Computation Cost No.Operation**	**Look-up** [**KB**]	**Table Size No.**
Partial Table Implementation			
[10]	33 × *A* + 1 × *DD*(3)	6.48	27 × 0.24
Speed	27 × *A* + 3 × *D*	54.00	18 × 3.00
V1	29 × *A* + 2 × *DD*(2)	15.84	18 × 0.88

Full Table Implementation			
[10]	33 × *A*	12.96	54 × 0.24
Speed	27 × *A*	162.00	54 × 3.00
V1	29 ×*A*	47.52	54 × 0.88

**Table 8.** Required number of finite field operations. Inv: inversion; Mul: multiplication; Sqr: squaring; overhead ratio: Inv = 8 × Mul, 4 × Sqr = 3 × Mul.

**Operation**	**Computation Cost**		
	**Inv**	**Mul**	**Sqr**
Addition	1	2	1
Doubling	1	2	2
Direct Doubling	1	(4r+1)	(4r+1)

**Table 9.** Evaluation result of fixed-base scalar multiplication under the pair condition (in terms of table size), where *A*, *D* and *DD*(*w*) denote addition, doubling and direct doubling (width), respectively. Computation cost excludes the cost for look-up table construction, which is pre-computable, so the cost mainly includes group addition and doubling.

**Op.**	**No. Op.**	**Inv**	**Mul**	**Sqr**	**Total**	**Size** [**Kbyte**]
Our size-optimized model						
SZ(V1)	28.9A+2DD(2)	30.9	75.7	46.9	357.8	15.8
SZ(V2)	28.9A+3DD(2)	31.9	84.7	55.9	381.5	15.8

Mohamed *et al.* (partial table implementation)						
2NAF	52.9A+DD(2)	53.9	114.8	61.9	591.5	3.2
3NAF	33.4A+DD(3)	34.4	79.7	46.4	389.5	6.5
4NAF	32.6A+DD(4)	33.6	82.3	49.6	388.5	12.8
5NAF	28.5A+DD(5)	29.5	78.1	49.5	351.6	25.6

Mohamed *et al.* (full table implementation)						
2NAF	52.9A	52.9	105.8	52.9	568.7	6.4
3NAF	33.4A	33.4	66.7	33.4	358.7	13
4NAF	32.6A	32.6	65.3	32.6	350.8	25.6
5NAF	28.5A	28.5	57	28.5	306.4	51.2

**Table 10.** Performance evaluation for the hybrid model in the case of 4NAF. V3 and V4 denote version 3 and 4, respectively. The bit section indicates the ratio of bits using our method and that of [10], respectively. Non-zero P: non-zero point.

**Bit**		**Non-zero P**		**Total**		**Size**[**KB**]	
**[10]**	**Here**	**V3**	**V4**	**V3**	**V4**	**V3**	**V4**
160	0	32.63	32.63	398.25	393.00	12.8	12.8
144	16	31.6	31.59	387.21	381.76	20.48	22.4
128	32	30.57	30.54	376.17	370.52	28.16	32
112	48	29.55	29.5	365.13	359.28	35.84	41.6
96	64	28.52	28.45	354.09	348.04	43.52	51.2
80	80	27.49	27.4	343.06	336.8	51.2	60.8
64	96	26.47	26.36	332.02	325.55	58.9	70.4
48	112	25.44	25.31	320.98	314.31	66.6	80
32	128	24.41	24.27	309.94	303.07	74.2	89.6

## Appendix. A

### Example of Proposed Method

In this section, we give a detailed process of proposed methods, including speed, size and hybrid models. Figure 4 shows the speed-optimized model. This model is constructed after two phases. First, Figure 1 is represented in *w*-NAF form using Algorithm 2; the corresponding output has the same structure as shown in Figure 3. Second, each element is grouped by window size (four). In our case, it is grouped in this order: [(*w*_0_, *w*_4_, *w*_8_, *w*_12_), (*w*_1_, *w*_5_, *w*_9_, *w*_13_), (*w*_2_, *w*_6_, *w*_10_, *w*_14_), (*w*_3_, *w*_7_, *w*_11_, *w*_15_), (*w*_16_,*w*_20_,*w*_24_, *w*_28_), (*w*_17_,*w*_21_,*w*_25_,*w*_29_), (*w*_18_, *w*_22_,*w*_26_,*w*_30_), (*w*_19_, *w*_23_, *w*_27_, *w*_31_), (*w*_32_,*w*_36_,*w*_40_,*w*_44_), (*w*_33_,*w*_37_,*w*_41_, *w*_45_), (*w*_34_,*w*_38_,*w*_42_,*w*_46_), (*w*_35_, *w*_39_,*w*_43_,*w*_47_), (*w*_48_, *w*_52_, *w*_56_, *w*_60_), (*w*_49_,*w*_53_,*w*_57_,*w*_61_), (*w*_50_, *w*_54_, *w*_58_, *w*_62_), (*w*_51_, *w*_55_, *w*_59_, *w*_63_)]. Afterward, then, scalar multiplication is conducted in the following order. Firstly, elements, including (*w*_3_, *w*_7_, *w*_11_, *w*_15_), (*w*_19_, *w*_23_, *w*_27_, *w*_31_), (*w*_35_, *w*_39_, *w*_43_, *w*_47_) and (*w*_51_, *w*_55_, *w*_59_, *w*_63_), are added to a point, and then, doubling is conducted. Secondly, elements, including (*w*_2_,*w*_6_,*w*_10_,*w*_14_), (*w*_18_, *w*_22_, *w*_26_,*w*_30_), (*w*_34_, *w*_38_, *w*_42_, *w*_46_) and (*w*_50_,*w*_54_,*w*_58_,*w*_62_), are added to the point, and then, doubling is conducted. Thirdly, elements, including (*w*_1_, *w*_5_, *w*_9_, *w*_13_), (*w*_17_, *w*_21_, *w*_25_, *w*_29_), (*w*_33_, *w*_37_, *w*_41_, *w*_45_) and (*w*_49_, *w*_53_, *w*_57_, *w*_61_), are added to the point, and then, doubling is conducted. Finally, elements, including (*w*_0_, *w*_4_, *w*_8_, *w*_12_), (*w*_16_, *w*_20_, *w*_24_, *w*_28_), (*w*_32_, *w*_36_, *w*_40_, *w*_44_) and (*w*_48_, *w*_52_, *w*_56_, *w*_60_), are added to the point.

For the size-optimized model in Figure 5, scalar multiplication value is represented in 3NAF form. Then, it is grouped in this order: [(*w*_0_, *w*_1_, *w*_44_), (*w*_2_, *w*_3_, *w*_46_), (*w*_4_, *w*_5_, *w*_48_), (*w*_6_, *w*_7_, *w*_50_), (*w*_8_, *w*_9_, *w*_52_), (*w*_10_,*w*_11_,*w*_54_), (*w*_12_,*w*_13_,*w*_56_), (*w*_14_,*w*_15_,*w*_58_), (*w*_16_,*w*_17_,*w*_60_), (*w*_18_,*w*_19_,*w*_62_), (*w*_20_,*w*_21_,*w*_64_), (*w*_22_,*w*_23_,*w*_45_), (*w*_24_,*w*_25_,*w*_47_), (*w*_26_,*w*_27_,*w*_49_), (*w*_28_,*w*_29_,*w*_50_), (*w*_30_,*w*_31_,*w*_53_), (*w*_32_,*w*_33_,*w*_55_), (*w*_34_, *w*_35_, *w*_57_), (*w*_36_, *w*_37_, *w*_59_), (*w*_38_, *w*_39_, *w*_61_), (*w*_40_, *w*_41_, *w*_63_), (*w*_42_, *w*_43_, *w*_65_)] Firstly, elements, including (*w*_2_, *w*_3_, *w*_46_), (*w*_6_, *w*_7_, *w*_50_), (*w*_10_, *w*_11_, *w*_54_), (*w*_14_, *w*_15_, *w*_58_), (*w*_18_, *w*_19_, *w*_62_), (*w*_22_, *w*_23_, *w*_45_), (*w*_26_, *w*_27_, *w*_49_), (*w*_30_, *w*_31_, *w*_53_), (*w*_34_, *w*_35_, *w*_57_), (*w*_38_, *w*_39_, *w*_61_) and (*w*_42_, *w*_43_, *w*_65_), are added to the point, and then, direct doubling by a width of two is conducted. Afterwards, then, elements, including (*w*_0_, *w*_1_, *w*_44_), (*w*_4_, *w*_5_, *w*_48_), (*w*_8_, *w*_9_, *w*_52_), (*w*_12_, *w*_13_, *w*_56_), (*w*_16_, *w*_17_, *w*_60_), (*w*_20_, *w*_21_, *w*_64_), (*w*_24_,*w*_25_,*w*_47_), (*w*_28_,*w*_29_,*w*_50_), (*w*_32_,*w*_33_,*w*_55_), (*w*_36_,*w*_37_,*w*_59_) and (*w*_40_,*w*_41_,*w*_63_), are added for completion.

For the hybrid model in Figure 11, scalar multiplication value is represented in 3NAF form. Then, it is grouped in two different orders. Firstly, elements from zero to 23 are constructed in this order: [(*w*_0_,*w*_1_,*w*_16_), (*w*_2_,*w*_3_,*w*_18_), (*w*_4_,*w*_5_,*w*_20_), (*w*_6_,*w*_7_,*w*_22_), (*w*_8_,*w*_9_,*w*_17_), (*w*_10_,*w*_11_,*w*_19_), (*w*_12_,*w*_13_,*w*_21_), (*w*_14_,*w*_15_,*w*_23_)]. Secondly, elements from 24 to 65 are grouped in this order: [(*w*_24_, *w*_25_, *w*_26_), (*w*_27_, *w*_28_, *w*_29_), (*w*_30_, *w*_31_, *w*_32_), (*w*_33_, *w*_34_, *w*_35_), (*w*_36_, *w*_37_, *w*_38_), (*w*_39_, *w*_40_, *w*_41_), (*w*_42_,*w*_43_,*w*_44_), (*w*_45_,*w*_46_,*w*_47_), (*w*_48_,*w*_49_,*w*_50_), (*w*_51_,*w*_52_,*w*_53_), (*w*_54_,*w*_55_,*w*_56_), (*w*_57_,*w*_58_,*w*_59_), (*w*_60_, *w*_61_, *w*_62_), (*w*_63_, *w*_64_, *w*_65_)]. After table construction, the scalar multiplication process is started. Firstly, elements, including (*w*_27_, *w*_28_, *w*_29_), (*w*_33_, *w*_34_, *w*_35_), (*w*_39_, *w*_40_, *w*_41_), (*w*_45_, *w*_46_, *w*_47_), (*w*_51_, *w*_52_, *w*_53_), (*w*_57_, *w*_58_, *w*_59_) and (*w*_63_, *w*_64_, *w*_65_), are added to the point, and then, doubling is conducted. Secondly, elements, including (*w*_2_, *w*_3_, *w*_18_), (*w*_6_, *w*_7_, *w*_22_) and (*w*_10_, *w*_11_, *w*_19_), are added to the point, and then, direct doubling by a width of two is conducted. Finally, elements, including (*w*_0_, *w*_1_, *w*_16_), (*w*_4_, *w*_5_, *w*_20_), (*w*_8_, *w*_9_, *w*_17_), (*w*_12_, *w*_13_, *w*_21_), (*w*_14_, *w*_15_, *w*_23_), (*w*_24_, *w*_25_, *w*_26_), (*w*_30_, *w*_31_, *w*_32_), (*w*_36_, *w*_37_, *w*_38_), (*w*_42_, *w*_43_, *w*_44_), (*w*_48_, *w*_49_, *w*_50_), (*w*_54_, *w*_55_, *w*_56_) and (*w*_60_, *w*_61_, *w*_62_), are added to the point.

## Appendix. B

### Proposed Method on an Unknown Point

Throughout this paper, we explored the proposed method on a fixed-point. However, we found that the method is also available for an unknown point. Ordinarily, *NAF_w_* representation is used for unknown point computation. By exploiting our structure, we can improve previous methods. The detailed structures of previous and proposed methods are described in Figures 18 and 19, respectively. This example shows the case of 32-bit input. First, the traditional method groups each element by a size of two in ascending order. Unlike the previous method, our method groups element in this order: [(*w*_0_, *w*_2_), (*w*_1_, *w*_3_), (*w*_4_, *w*_6_), (*w*_5_, *w*_7_), (*w*_8_, *w*_10_), (*w*_9_, *w*_11_), (*w*_12_, *w*_14_), (*w*_13_, *w*_15_), (*w*_16_, *w*_18_), (*w*_17_, *w*_19_), (*w*_20_, *w*_22_), (*w*_21_, *w*_23_), (*w*_24_, *w*_26_), (*w*_25_, *w*_27_), (*w*_28_, *w*_30_), (*w*_29_, *w*_31_)]. This structure shows a higher zero occurrence ratio than previous method, and its result is available in Table 5. After table construction, the scalar multiplication process is started. Firstly, element (*w*_0_, *w*_2_) is added, and then, one doubling process is conducted. Secondly, element (*w*_1_, *w*_3_) is added, and then, direct doubling by three is conducted. This process is repeated, until the end of computation.

In the Table 1, we compared the size of the look-up table and the computation costs of previous and proposed methods on an unknown point in the case of 160-bit scalar multiplication with 2NAF representation. In terms of size, the previous method has results for (10) and (01), and the proposed method has results for (101), (100), (10-1) and (001). Each result has 40 byte values, including X and Y values, so the previous method occupies 80 bytes and ours occupies 160 bytes. For look-up table computation, one doubling is required for the traditional method, to compute (10). In the case of ours, two additions and a direct doubling by two are needed to compute (101), (100) and (10-1). After look-up table computation, point computation is conducted. By exploiting high zero occurrence, our method shows a small number of addition computation and our method conducts a doubling and a direct doubling by three. Total computation costs, including pre-computation and point-computation, are listed in the Table 1. Our method shows speed performance enhancement by 12.7% compared to traditional *NAF*_2_ representation for an unknown point.
